# Refugee families in Iceland: opportunities and challenges in schools and society

**DOI:** 10.1080/17482631.2020.1764294

**Published:** 2020-12-09

**Authors:** Hanna Ragnarsdóttir

**Affiliations:** School of Education, University of Iceland, Reykjavík, Iceland

**Keywords:** Syrian quota refugee families, children with refugee background, preschools, compulsory schools, educational practices, home–school communication

## Abstract

In 2016, a group of 55 Syrian quota refugees arrived in Iceland from Lebanon and settled in three municipalities. There were 11 families comprising 20 adults and 35 children. This study^1^ aimed to critically explore the experiences, opportunities and challenges of these children, their parents, their teachers and principals in the municipalities of their resettlement since their arrival in Iceland. The theoretical framework of the study includes critical approaches to education, and multilingual education for social justice. Methods of data collection included semi-structured interviews with the refugee parents, the head teachers and teachers in all the schools in the study. While the findings indicate that most of the children were doing well both academically and socially in their first months in the schools, they also show that the children and parents have experienced a number of challenges. These included illiteracy, interrupted schooling of the children and hidden trauma before arriving in Iceland. After arrival, the parents have experienced lack of communication between schools and homes, as well as differences in norms, values, languages, and expectations between the schools and homes.

## Introduction

Ethnic and linguistic diversity has been steadily growing in Iceland in recent years as a result of growing migration to Iceland. While foreign citizens in Iceland were 1,8% of the total population in 1995, the percentage reached 10,9% in 2018 (Statistics Iceland, 2019a). Statistics Iceland defines an immigrant as a person born abroad who has both parents and all grandparents foreign born (Statistics Iceland, 2019b). The diversification of the population is reflected in the education system, where, in 2017, around 12% of all preschool children and 10% of all compulsory school students had other first languages than Icelandic (Statistics Iceland, 2019c, 2019d). Currently, 17,010 people whose country of birth is Poland live in Iceland, which makes Polish by far the largest group of immigrants. Smaller groups include, for example, Philippines born, 1,878 people and Syrian born, 217 people (for more detail, see Statistics Iceland, 2019e). The numbers of religious and life stance organizations in Iceland have also grown in recent years, reflecting the increase in ethnic diversity (Statistics Iceland, 2019f). Immigrants to Iceland include resettled refugees. Altogether 778 refugees have resettled in Iceland in the past decades, from 1956 to 2019 (Government of Iceland, n.d.). They are invited to Iceland by the government and settle in various municipalities in the country. Before the refugees arrive, decisions regarding from which countries refugees arrive are made in cooperation with the UN Refugee Agency (United Nations High Commissioner for Refugees, UNHCR).

In 2016, a group of 55 Syrian quota refugees arrived in Iceland from Lebanon and settled in three municipalities (Government of Iceland, n.d.). The group comprised 11 families; 20 adults and 35 children. This study aimed to critically explore the experiences, opportunities and challenges of these children, their parents, their teachers and principals in the municipalities of their resettlement since their arrival in Iceland.

## Background and context

The guidelines for the reception of quota refugees, i.e., refugees invited to resettle in Iceland (Stjórnarráð Íslands [Government of Iceland], 2013) stipulate that services are provided for 1 year after the arrival of the refugees. The Minister of Welfare oversees the reception according to the guidelines. The role of the refugee committee of the Icelandic government, including representatives from the Ministry for Foreign Affairs, the Ministry of the Interior, the Ministry of Welfare and the Icelandic Red Cross, is to decide in cooperation with the UNHCR from which country or countries refugees should be invited each year. The committee then proposes to the government of Iceland a general policy and organization of the reception of refugee groups each year. Based on this proposition and a cost estimate, the Minister of Welfare and the Minister for Foreign Affairs propose to the government of Iceland that a certain number of refugees from a certain country or countries should be invited to resettle in Iceland. The government makes a final decision regarding the numbers of refugees based on the proposition. The Icelandic Red Cross cooperates with the government in finding three to five support families for each refugee family. A contract is made with certain municipalities for receiving and providing services for refugees in accordance with the decision made by the government each time. Certain criteria are considered when the municipalities are chosen, including the availability of social services, health services, jobs, possibilities for education, housing and other matters considered to be of importance in each case. The refugees are granted health services, courses in Icelandic, courses on Icelandic society, first (L1) language teaching where possible, leisure activities, services of interpreters, assistance in finding jobs and other necessary services. Each municipality prepares for the arrival of the families assigned to its area. Following a medical examination, the children start school. This is normally a few weeks after arrival (Icelandic Red Cross, n.d.).

Educational policies and curriculum guides in Iceland emphasize equity and inclusion (Ministry of Education, Science and Culture, 2011, 2016). This entails that schools are expected to provide all pupils with appropriate access and opportunities, irrespective of sex, economic status, geographic location, religion, disability, and cultural or social background. Schools are required to have reception plans for pupils whose first language is not Icelandic. These pupils have the right to learn Icelandic as a second language. According to the Icelandic Compulsory school Act (no. 91/2008), the aim of the training shall be that the pupils become actively bilingual and ready for the regular compulsory school as well as preparing them to become active citizens in Icelandic society. The teaching of first languages is not stipulated in the acts, but this has been applied in schools where possible. While the Icelandic Preschool Act (no. 90/2008) emphasizes a whole child approach, it does not include a section on first languages. Education (primary and lower-secondary education) in Iceland is compulsory between the ages of 6 and 16 and strong emphasis is also placed on upper-secondary education. Municipalities in Iceland are responsible for the operation of preschools and compulsory schools and schools must develop their own curricula based on the national curriculum guidelines (Ministry of Education, Science and Culture, 2016).

According to the guidelines for the reception of quota refugees in Iceland (Stjórnarráð Íslands [Government of Iceland], 2013), children with refugee background should, in addition to learning Icelandic, have teaching in and through their first languages where possible, thus aiming for the active bilingualism of these children. For how long pupils are entitled to instruction in L1 is not stipulated. The children should have the assistance of an interpreter or first language teacher in schools. Before the children attend schools, the staff and students in the schools should be provided with basic knowledge of their background and conditions in their countries of origin. Generally, the aim of the reception and support is to enable as well as possible the integration of the refugees into Icelandic society.

## Theoretical framework

The theoretical framework of the study includes critical approaches to education (May & Sleeter, 2010), and theories about multilingual education for social justice (Cummins, 2004). These approaches provide an important framework with which to critically address the educational experiences of the children and students. Writings on challenges and initiatives in educational settings (Block et al., 2014; Ferfolja & Vickers, 2010; Glover & Hemingway, 2005; Keddie, 2012; Ragnarsdóttir & Kulbrandstad, 2018; Ragnarsdóttir & Schmidt, 2014) and writings on the well-being of children with refugee background are also consulted (Alsayed & Wildes, 2018; Khamis, 2019; Rousseau et al., 2012; Ziaian et al., 2012).

### Critical approaches to education

Various critical approaches highlight the position of minority groups in schools and societies from a critical perspective and analyse what factors in educational and societal structures cause and maintain unequal status (May & Sleeter, 2010). Banks (2013) claims that educational systems need to critically address inequalities and ensure equality, social justice, empowerment, voice, and dialogue for their individual students and teachers. Similarly, Cummins (2004) claims that inequalities related to children’s diverse languages exist in schools, in that some educational approaches towards children’s diverse languages can include some students and exclude others. In order to create learning spaces that respond to the needs of linguistically and culturally diverse groups of students, schools need to consider how to implement socially just and inclusive practices that build on students’ prior knowledge and welcome diverse identities and backgrounds (Cummins, 2001).

### Challenges and initiatives in educational settings

Researchers (Block et al., 2014) have claimed that with the increase of refugees and asylum seekers worldwide, there is a growing recognition of the importance of the school environment for promoting successful settlement outcomes and including young refugees. However, some schools are poorly equipped to recognize and respond to the various challenges that children with refugee background face, such as learning a new language while grappling with unfamiliar educational and social systems. Research has indicated that cultural mismatch in the school context and home context may lead to lack of communication and misunderstanding between schools and homes (Ragnarsdóttir, 2008).

Many researchers have documented initiatives in schools at different levels where inclusive learning spaces and leisure time environment are developed for diverse groups of students, including refugees (Ferfolja & Vickers, 2010; Glover & Hemingway, 2005; Keddie, 2012; Ragnarsdóttir & Kulbrandstad, 2018; Ragnarsdóttir & Schmidt, 2014).

### Well-being of children and families with refugee background

Findings of research related to children and families with refugee background have indicated that many refugees have experienced difficult situations that can affect their daily life and education. Challenges in resettling in a new country include differences in values, norms and languages (Jensdóttir, 2016) and dealing with mental health problems (Ziaian et al., 2012). Reporting on their research with young refugees resettled in South Australia, Ziaian et al. (2012) discuss that if refugee parents are themselves psychologically distressed, they may be less aware of their children’s problems.

Emphasizing the strengths and resilience of children with refugee background is also important. Alsayed & Wildes (2018), reporting on their study of strengths and difficulties of Syrian children with refugee background in Turkey, emphasize that schools should address both the difficulties and the strengths of refugee children. They point out that large numbers of war-affected children exhibit remarkable resilience and actively cope with, adapt to, and navigate complex situations of adversity. In their research, the children enjoy play, want to discuss their situation and hold opinions about their needs.

Refugee families face various challenges when resettling in a new country, including adjusting to new conditions and new roles. According to Richman (1998), this can lead to a loss of role, respect, and status that may result in depression or other ailments. While many refugees are well educated and have skills and professions that could contribute to their community, research in Iceland has indicated that their qualifications are rarely recognized (Alþjóðamálastofnun Háskóla Íslands [Institute of International Affairs], 2017). This can cause difficulties and depression for many refugees. Additionally, according to Richman (1998), social networks are often lacking and therefore, many women and mothers of young children may experience isolation when they resettle in a new country.

## Method

A qualitative, in-depth approach was chosen to conduct the research and to understand the experiences and views of the participants (Denzin et al., 2008; Flick, 2006).

In this research, establishing respectful relationships according to culturally responsive methodologies (Berryman et al., 2013) with participants from the beginning was prioritized. An Arabic-speaking co-researcher and doctoral student, herself a refugee and therefore familiar with the experiences of resettling established contact with the refugee parents. She had previously worked for the Red Cross in the reception of refugees in Iceland. The co-researcher was present in all the interviews with the parents and conducted the interviews in Arabic. During the interviews conducted in English, she interpreted when the parents chose to switch over to Arabic. The parents chose the language of the interviews and the researchers prepared according to the parents’ choice. The emphasis in all interviews was on creating an environment of trust and gaining in-depth data.

The participants were quota refugee parents in 11 families, as well as head teachers and teachers in six preschools and four compulsory schools in three municipalities. There were altogether 20 adults and 35 children in these 11 families and most of these children attended preschools and compulsory schools at the time of the research. Permissions were sought in the central educational offices of the three municipalities and the head teachers of the children’s preshools and compulsory schools gave their permission for conducting interviews in the schools. Information about the families and children were obtained. The Arabic-speaking co-researcher contacted the parents informally and invited them to participate, and they all accepted.

All the participating parents in the study are of Syrian nationality and stated Arabic as their first language. The education of the participants differed. Three of the parents (mothers) had short or no basic schooling and were illiterate in Arabic, while five of the parents (three fathers and two mothers) had finished higher education and spoke and read English in addition to Arabic.

The project followed the general ethical practices for research involving humans: respect of the rights, interests and dignity of the participants and related persons (Kvale, 2007). The Icelandic Data Protection Authority was informed of the research, and it was carried out in accordance with the University of Iceland Scientific Ethical Guidelines (University of Iceland, 2014). An informed consent form was developed in Arabic and presented to the parents and an informed consent form in Icelandic was presented to the school staff. Their anonymity was ensured, and their decisions on language and location of the interviews were respected during the entire study period.

Methods of data collection included semi-structured interviews with the refugee parents as well as with the head teachers and teachers in all the schools in the study. The interviews were conducted in the period from autumn 2016 to summer 2017. Semi-structured interviews were chosen to collect data as they are considered one way of establishing respectful relationships with participants in close proximity to them and obtaining in-depth data (Kvale, 2007). Semi-structured interview guides were developed before the interviews. However, during the interviews, the researchers carefully provided both time and flexibility to allow the participants to talk freely about issues of their choice that were not covered in the interview guides.

The interviews with the refugee parents took place in the participants’ homes. The families had been in Iceland from 9 months up to 1 year at the time of the interview. Both parents of each refugee child participated together in the interviews, except in one case of a single parent. The interviews were conducted in English with parents who spoke English and in Arabic with parents who did not speak English. The co-researcher who speaks Arabic conducted the interviews in Arabic. The interviews lasted on average an hour.

The interviews with the school staff took place in the six preschools and four compulsory schools and were in Icelandic. All the head teachers were interviewed and they were asked to suggest a second participant from their school, who had worked closely with the refugee child or children. Fourteen interviews were conducted with altogether 23 of the staff in these schools, thereof eight head teachers, five heads of divisions, six supervisory teachers and four special educators.

The interviews were recorded and transcribed verbatim. The qualitative procedures of content analysis were applied for analysing the interviews, including coding and constant comparison of data (Flick, 2006). The researchers read and re-read the transcripts. First, codes were developed and then the researchers discussed the codes and developed main themes based on these codes. The interviews that were conducted in Arabic were transcribed in Arabic. Excerpts from the interviews in the findings chapter which were in Icelandic and Arabic were translated into English.

## Findings

This section presents findings from the interviews with the parents as well as the head teachers and teachers in the children’s schools. Themes drawn from the data and presented below are as follows: *Reception of refugee families and first months in a new country; educational practices in preschools and compulsory schools*; and *educational partnerships between parents and schools.*

### Reception of refugee families and first months in a new country

Many of the participants noted how important the support of the community and the support families had been, both for the schools and the families. In the smallest of the municipalities, people’s views towards the refugees were very positive, according to the staff of the schools. Perhaps this was related to the small size of the place and close personal relationships.

While the schools in the study had all worked with ethnically and linguistically diverse groups of children and students, including immigrant students, before the arrival of the children with refugee background, none of these schools had previously received refugees. The refugee families arrived at slightly different times in 2016. Since both the children and their parents were eager for them to start school, the preparation period was shorter than had been planned. One of the compulsory school teachers noted that:

These children are hungry for education. It is just wonderful. I have not experienced anything like this before. They are so diligent, and they want so much to learn … they have waited for so long.

The participating staff in the schools generally noted that they were very content with the preparation organized by the municipalities. The preparation included several meetings with the school leaders, other meetings for the entire school staff, and short courses on various issues related to refugees, such as trauma and different aspects of teaching children with refugee background. Different individuals were responsible for these courses, including the project managers of refugees and asylum seekers from the Red Cross and specialists like psychologists.

However, some of the teachers claimed the support from the central educational offices had lasted for too short time and that they were later left alone to “deal with the task.” Despite the general good preparation at the central educational offices which the teachers described, they felt that when the children arrived they knew very little about them individually. One of the teachers noted,

When they arrived, we knew nothing about them. We knew nothing about their status, so we could not organize how we should do this … but we soon put the younger children into classes.

Although the children and parents expressed their gratitude for the reception and support which they received in Iceland, most of the parents were already disappointed soon after arriving in Iceland. They had expected more financial and social support and better educational support for their children. One of the parents noted,

Our situation was the same as in Lebanon. It is not better now. They didn’t do what they promised to do. They informed us before we came here that there would be at least one teacher or someone who will assist our daughters and there would be different kinds of support both academic and financial for our kids. They are so upset that one of them came home from school yesterday and told me that she wants to go back to Lebanon or Syria. If you see the situation of immigrants in Canada and in Germany, they live much better and get more support than us.

Many of the teachers noted how different the conditions of the families were, some had, for example, experienced conflict while others had not. Nevertheless, the teachers claimed that the families all needed support in adapting to the new conditions and climate and many of the teachers noted how the parents’ feelings fluctuated during the first year. The teachers showed understanding for how difficult it was for the families to adapt to such different conditions. One teacher felt sorry for a young refugee mother who she thought was very isolated at home:

… it must be a very strange life and of course … Icelandic parents are very bored to stay alone at home with a young baby. … and how this woman is managing, alone in a foreign country where there is darkness almost all day and now light the whole day and night.

The staff of the schools generally showed understanding and claimed that they tried their best to support the refugee families and children, while many of them deplored the lack of follow-up support from the municipalities after the initial preparation period.

### Educational practices in preschools and compulsory schools

The participating schools differ in many ways. Two of the schools have extensive experience of working with ethnically diverse groups of children and families, while other schools do not have such experience. A preschool teacher noted how different it was to receive children with refugee background from receiving immigrant children, particularly in building trust. She said,

Additionally, there is the shock that the children … and also parents, have experienced … they found it very difficult to trust … and wondered whether the gate was carefully locked and so on … we have to convince them that he (the child) is safe here.

The schools used different methods to develop trust and understanding of the children with refugee background and their parents. Two of the preschools used books for communication, including pictures and Icelandic words, and sending these books home with the children. The parents put pictures of themselves and their children in the books and wrote words in Arabic. This helped both parents and schools to communicate, share experiences, languages and develop trust. One preschool head teacher noted,

They were very conscientious to always bring the book to the preschool, every morning. They said they also used it to learn Icelandic.

The preschool teachers reported using many methods to facilitate the children’s learning of Icelandic as a second language, while at the same time making all first languages of the children in the preschools visible and using these for singing. They said this was also a strategy for empowering the children, developing trust and security.

The refugee preschool children were generally doing well according to the preschool teachers, who noted that the parents also seemed to be satisfied with the preschools.

The children attending the lower grades of the compulsory schools generally took part in all activities with their peers in their classes, while also receiving extra support in learning Icelandic as a second language (ISL). The extent of this extra support depended on each refugee child’s needs. The teachers in the participating schools maintained that they organized the children’s curricula based on their evaluation of the children’s educational position and need for support. One of the compulsory school teachers said,

We first received two boys, and I wanted the school to agree to let them participate in the classes from the beginning if their position was such that they could handle it at least partly … and they were tested. These were boys who had been in school before. They had a certain basis, so we put them directly into classes in math, English, science, social science … physical education and swimming. And they took Icelandic lessons with me.

The compulsory school teachers claimed that the older children experienced the greatest difficulties, including learning ISL and some other subjects, and some were socially isolated from their peers, while the younger children generally showed quicker progress. A compulsory school teacher noted,

You can feel it. You experience that the younger children are much stronger because they achieve these social contacts sooner than the older children. They are all doing very well, at least I think so. Unbelievable progress after such a short time.

The refugee parents generally talked about good first experiences of their children’s preschools. One parent of a preschool child noted,

I went to the kindergarten with my other two kids. It was very good. They understand us, they know that we came from war.… The head teacher gave the students some idea about who we are, where we came from. He gave the kids some information about us. We found that they care, and they understand us.

Refugee parents of compulsory school children experienced more challenges. Parents in three of the families were concerned about the lack of information from the schools, while the teachers said that they were trying their best to cope with communicating across languages, using all the methods they could think of in their daily communication. Interpreters were always used in formal meetings with the parents. However, the parents who had higher education talked about the lack of services in the schools to facilitate their children’s adjustment and learning. One parent mentioned a support person who had little knowledge of the Icelandic language and could not assist their children. The parent noted,

You know, we have five children at school. The main reason behind moving here was to educate them, and we want them to learn. Over there in Lebanon, we had no chance to send them to school. We don’t care whether the school hired her or not but what we need is someone who can help and support our children, not someone who only sends and receives messages. We can translate things by ourselves through Google translate or ask others to help us.

The refugee parents who had higher education complained about unclear grading practices and inappropriate assessment and noted that this did not help them to understand how their children were progressing in their studies. They also had doubt about the quality of education in Iceland and whether this would benefit their children. One parent said,

I’m concerned about their future as well as their present. What do they benefit from being here in Iceland, what do they learn, what do they get, are they in the right place, there is a question mark, I don’t know what they learn, what they take from the society and there will be a question mark.… There are things that are clear concerning schools, like mathematics and reading but still we find a big gap between us and the system, the kids even feel that there is a gap. We hope that this will change in the future when they learn more Icelandic.

The refugee parents talked about the need for an interpreter or someone who had adequate knowledge in their language and the municipality of their resettlement to assist them and their children. The parents of the compulsory school children worried that their children would be marginalized since they didn’t understand Icelandic and could not take the exams. They wanted their children to be able to take the tests in Arabic.

To summarize, although parents’ and teachers’ experiences of the first months of schooling were in many ways positive, both parents and teachers talked about numerous challenges related to the children’s schooling. Some of these included lack of communication as well as different expectations and school cultures.

### Educational partnerships between parents and schools

The school staff were very pleased with the cooperation with the refugee parents.

Although the families differ in many ways as noted above, the cooperation was generally good according to the staff. They maintained that good preparation had facilitated this. The language abilities of the refugee parents differed immensely as previously noted. Translations of messages into Arabic and using digital tools to communicate did not help the parents who were illiterate. However, in spite of illiteracy, the communication was generally good according to the teachers and head teachers.

As the first months passed, the worries and disappointments of the refugee parents increased according to the school staff, who talked about highs and lows in the parents’ feelings and that they had been prepared for this.

A preschool teacher said,

I think the shock is coming to them now. First when they arrived they were extremely grateful … they were so happy and excited and ready to learn and so on. Now I feel they are rather bitter … and they (say that they) do not get as much as the other family. Less is being done for them and so on. Yes, I experience sadness and lack of trust.

Experiences of isolation were also expressed by the parents. One parent said,

There is no social life here, sometimes I ask my husband why it is like this … There are for example, two kids the same age of my son … but they live far away. I want to communicate but sometimes (there is) no response from the other side. We only visit the family … they came with us and they are close. Our kids stayed there one night and they loved it. Others are far away. They put us in different places.

The parents also found it difficult to take time to participate in social activities because of limited time. One parent said,

Well, we find it difficult, we don’t work but we still find it difficult to make time for everything. When they come home we eat our lunch, they go to training. When they come home I have to give them a bath. I have to teach them. You know our timetable is full. We don’t know for example, … I want to take them to the cinema but I don’t know how. I want to take them to the zoo, I don’t know how.… We want to go out to eat, we don’t know where to go. We want our children to feel the life here but we don’t know many things, many places.

The findings indicate that there is a discrepancy between the perspectives of some parents and perspectives of some of the teachers. Parents in two of the families were concerned about the lack of cultural awareness and understanding of the teachers, and that this could impede teaching as well as collaboration between home and school. One of them said,

I think that they should know about the basic principles of our culture and religion, and also about our festivals. In general, the more you know about your students, the better you will understand and be engaged with them in the teaching-learning process.

Parents and children who had some education in Syria realized that the differences between the school systems in Syria and in Iceland presented challenges for them as well as their teachers. Some of the topics or lessons conflicted with their religious values and cultural norms, such as mixed-gender swimming classes and the teaching of different religions. Several teachers described the challenges related to cultural and religious differences and how they assumed that these affected relations between the children, particularly in the case of the adolescent girls. In one of the compulsory schools, one of the teachers noted,

They have become a little sad because they are so isolated … socially.… They wear a hijab and stood out from the whole group when they first arrived.

Her colleague discussed the differences between the adolescent boys and girls. Referring to boys who arrived a year earlier in another group of quota refugees and comparing these boys with the girls she said,

We see a great difference. The boys who came here two years ago … who were in the seventh and tenth grade, they got a bus card and they went all over town. They even got lost, but they found their way back home. The girls don’t go. They don’t even go down to the town.… So, there is a big difference between them.

The first teacher described how they had tried to involve the girls in various social activities, without success. She noted that she had considered cultural and religious differences and how to support the girls in participating. Discussing her experiences of religious differences in other countries and how girls were expected to stay at home, she added,

In our case, it is not that the family wants them to stay at home. In fact, their mother worries that they stay at home too much and have no friends.

The teachers in this school noted that the girls somehow were reluctant to go out and participate, and worried about their isolation from their peers and their future in Icelandic society.

## Discussion and conclusion

The aim of this study was to critically explore the experiences, opportunities and challenges of these refugee children, their parents, their teachers and principals in the municipalities of their resettlement since their arrival in Iceland.

Refugee families, who have been invited to Iceland, resettle under organized circumstances, where municipalities and schools are expected to adhere to the guidelines for the reception of quota refugees (Stjórnarráð Íslands [Government of Iceland], 2013). Furthermore, schools are expected to base their work on principles of equity and inclusion (Ministry of Education, Science and Culture, 2011, 2016). In spite of these privileged circumstances, many challenges appear in the process of resettling.

Scholars (Hamilton & Moore, 2004; Khamis, 2019) emphasize that schools play a key role in children’s well-being, as well as facilitating socialization and children’s adjustment to a new context. While the findings of this study indicate that most of the children were doing well both academically and socially in their first months in the schools, both the children and their parents have experienced a number of challenges. The teachers particularly talk about the challenges facing the older children, both academically and socially. These include illiteracy, having missed out on education for some years, and starting to learn a new language in adolescence. While the children go through medical examination before starting school, the schools have little information about the children’s educational background and assessment tools for migrant and refugee children are not well developed. Support is also lacking in Arabic. No teacher in the study spoke both languages and an Arabic teacher provided teaching outside the school only in one of the municipalities. The schools are therefore not able to follow the guidelines of supporting the refugee children’s first language. The compulsory schools included in this study did not actively build on the children’s first language and multilingual education, as introduced by Cummins (2001, 2004), is generally not in place in these schools. The emphasis is on teaching Icelandic as a second language; schools do not generally provide teaching and resources in the children’s first language. However, two of the preschools work with diverse languages in different ways in their everyday practices, including Arabic. With a few exceptions, teachers generally have no training in multilingual education (Cummins, 2004). The children would benefit from multilingual approaches as well as inclusive practices that build on their prior knowledge (Cummins, 2001). Furthermore, an understanding of the diversity within the group of quota refugee families seems to be lacking among the teachers, resulting in some misunderstanding and preconceptions about the parents and children.

The findings of the research conducted with the teachers and principals indicate that there is lack of follow-up support for the schools from the municipalities. Furthermore, an emphasis on community building, as Alsayed & Wildes (2018) have suggested, has only developed in one of the municipalities. In others, the parents experience isolation. Although the parents talk about the relief of coming to a peaceful country, they also express their loss of social networks and family, structural discrimination and loss of role and status (Richman, 1998). As noted in the findings, most of the parents have middle or higher education. They have skills and professions that could contribute to their community, but they deplore that their qualifications are not recognized in the new country. Furthermore, many of the women in the group face new situations and responsibilities in Iceland. Two of them have lost their roles as independent working women while others who were not used to going out alone have begun working outside their homes. Additionally, some of the women and mothers of young children experience isolation in Iceland.

The refugee parents in the study are aware of the importance of education in their children’s lives and want them to learn quickly. However, they experience challenges related to differences in expectations and school culture. This is particularly evident among the highly educated parents in the study. Block et al. (2014) noted that schools may be poorly equipped to recognize and respond to the multiple challenges which children and young people with refugee background face. The staff in the schools believe that they have generally cooperated actively with parents, while some of the parents deplore that they lack information from the schools. This is evident among parents with different education, both higher education and little education. Furthermore, some parents, particularly those who are highly educated, worry that their children are not learning enough and not quickly enough. The teachers are concerned about illiteracy among both the children and parents and that some of the children have missed some years of schooling. The teachers and head teachers generally show understanding towards and care for the refugee parents and children.

Although the staff of the schools are prepared for and have been trained for children’s traumas in the preparatory courses held in the municipal central offices, trauma does not seem to play a major role for the refugees in the study, while conditions in Iceland and a mismatch in expectations appears to be more important. The lack of hope and future prospects also seems to play a part. However, some of the teachers talk about highs and lows appearing in the parents’ feelings, the initial gratitude followed by later disillusionment. The teachers also worry about latent traumas, which may affect the lives of the children (Richman, 1998; Rousseau et al., 2012; Ziaian et al., 2012), and give examples of children’s play that suggested possible experiences of violence. However, the data does not indicate the reason for the violence in the children’s play. Rather, the parents talk about the need for linguistic and academic support for their children and for an understanding of their culture. Their initial gratitude and excitement have been replaced by anxiety and doubt as they worry about their children’s education and future.

To conclude, according to the findings, the children would benefit from multilingual approaches in their schools and the parents would benefit from more active educational partnerships with the schools and community building in helping them establishing a better life in Iceland.1

## Note

1. This study was conducted by Hanna Ragnarsdóttir and Susan Rafik Hama, School of Education, University of Iceland.
